# Increase in Acute Flaccid Myelitis — United States, 2018

**DOI:** 10.15585/mmwr.mm6745e1

**Published:** 2018-11-16

**Authors:** Susannah L. McKay, Adria D. Lee, Adriana S. Lopez, W. Allan Nix, Kathleen L. Dooling, Amelia A. Keaton, Emily Spence-Davizon, Rachel Herlihy, Thomas A. Clark, Sarah E. Hopkins, Daniel M. Pastula, James Sejvar, M. Steven Oberste, Mark A. Pallansch, Manisha Patel, Janell A. Routh

**Affiliations:** ^1^Epidemic Intelligence Service, CDC; ^2^Division of Viral Diseases, National Center for Immunization and Respiratory Diseases, CDC; ^3^Division of Foodborne, Waterborne, and Environmental Diseases, National Center for Emerging and Zoonotic Infectious Diseases, CDC; ^4^Colorado Department of Public Health and the Environment; ^5^National Center for Immunization and Respiratory Diseases, CDC; ^6^Division of Neurology, Children’s Hospital of Philadelphia, Pennsylvania; ^7^University of Colorado School of Medicine, Aurora, Colorado; ^8^Division of High-Consequence Pathogens and Pathology, National Center for Emerging and Zoonotic Infectious Diseases, CDC.

In August 2018, CDC noted an increased number of reports of patients having symptoms clinically compatible with acute flaccid myelitis (AFM), a rare condition characterized by rapid onset of flaccid weakness in one or more limbs and spinal cord gray matter lesions, compared with August 2017. Since 2014, CDC has conducted surveillance for AFM using a standardized case definition (*1*,*2*). An Epi-X* notice was issued on August 23, 2018, to increase clinician awareness and provide guidance for case reporting.

Patients who meet the clinical case criteria for AFM, defined as acute flaccid limb weakness, are classified using the Council of State and Territorial Epidemiologists case definitions of “confirmed” (magnetic resonance imaging [MRI] with spinal cord lesion largely restricted to gray matter and spanning ≥1 spinal segments), “probable” (cerebrospinal fluid [CSF] pleocytosis [>5 white blood cells per mm^3^]), or “not a case.”

Among 106 patients with acute flaccid limb weakness classified during January 1–November 2, 2018, 80 cases of AFM were classified as confirmed (from 25 states) (Figure), 6 as probable, and 20 as noncases. This represents a threefold increase in confirmed cases compared with the same period in 2017. Among confirmed cases, the median patient age was 4 years (range = 7 months–32 years; interquartile range [IQR] = 2.4–7.6 years), 47 (59%) were male, and, among 65 patients with information on race available, 56 (86%) were white. During the 4 weeks preceding the onset of limb weakness, signs and symptoms consistent with a viral illness were reported for 79 (99%), including fever for 65 (81%), respiratory symptoms (e.g., cough, rhinorrhea, and congestion) for 62 (78%), and gastrointestinal symptoms (e.g., vomiting and diarrhea) for 30 (38%) patients with confirmed AFM. Upper limb only involvement was reported by 38 (47.5%) patients, lower limb only by 7 (8.8%), two to three upper and lower limbs by 12 (15.0%), and all four limbs by 23 (28.8%). All patients with confirmed AFM were hospitalized, and 47 (59%) were admitted to intensive care units; no deaths have been reported.

**FIGURE:**
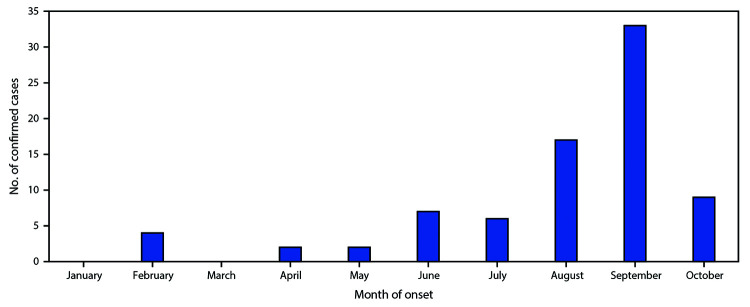
Number of confirmed cases of acute flaccid myelitis (AFM) reported to CDC, by month of onset — United States, January–October 2018* * Confirmed AFM cases that CDC was made aware of as of November 2, 2018. Patients under investigation are still being classified, and the case counts are subject to change.

Among 78 (98%) confirmed cases with available CSF results, 65 (83%) had pleocytosis, with a median cell count of 103 cells per mm^3^ (range = 6–814; IQR = 56–194); most had a lymphocyte predominance. Median CSF protein and glucose were 47 mg per dL (range = 8–289; IQR = 37–62; normal <45) and 59 mg per dL (range = 40–138; IQR = 52–65; normal ≥40), respectively. The median interval from limb weakness to CSF collection was 1 day (range = 0–16; IQR = 1–3). The median interval from sign or symptom onset to CSF collection was 7 days (range = 0–23; IQR = 5–8) for respiratory illness, 4 days (range = 0–22; IQR = 3–7) for gastrointestinal symptoms, and 3 days (range = 0–17; IQR = 2–6) for fever.

CDC conducts enterovirus/rhinovirus (EV/RV) testing for all patients meeting the clinical criteria for AFM, when specimens are available. Of the 80 confirmed cases in 2018, testing was performed on a total of 125 clinical specimens from 71 (89%) patients, including 21 CSF, 59 upper respiratory, and 45 stool/rectal swab specimens (Table). Among these, specimens from 38 (54%) patients were positive by EV/RV real-time reverse transcription–polymerase chain reaction testing, including 11 (29%) for EV-A71, 14 (37%) for EV-D68, and 13 (34%) for other viruses, primarily from nonsterile sites. CSF specimens from two patients were positive. One CSF specimen was positive for EV-A71; this patient also had a stool specimen positive for EV-A71. The second patient had a CSF specimen positive for EV-D68; this patient also had EV-D68 and parechovirus-A6 identified in a respiratory specimen. Two additional patients had more than one virus detected in a single respiratory specimen, including one with EV-D68 and echovirus 6 and one with RV-A24 and parechovirus-A6. All stool specimens tested negative for poliovirus. Among the 20 patients who did not meet the AFM case definition and were classified as non­cases, 1 (5%) had a positive CSF specimen (echovirus 25), 7 (35%) had positive respiratory specimens (EV-A71, RV-A24, RV-A56, RV-A90, EV/RV not typed), and 6 (30%) had positive stool or rectal swab specimens (EV-D68, EV-A71, RV-A90, echovirus 9, echovirus 11, echovirus 25).

**TABLE:** Enterovirus/rhinovirus (EV/RV) type testing results* of specimens from patients with confirmed acute flaccid myelitis and specimens positive for EV/RV, by specimen type — United States, January–October 2018

Enterovirus and rhinovirus testing, by type	CDC laboratory results			Total (N = 125)
	CSF specimens (n = 21)	Respiratory specimens (n = 59)	Stool/Rectal swab specimens (n = 45)	
**EV- or RV-positive no. (%)**	**2 (10)**	**31 (53)**	**17 (38)**	**50**
**Subtype, no. (%) positive^†^**				
EV-A71	1 (50)	10 (32)	10 (59)	21 (42)
EV-D68	1 (50)	13 (42)	1 (6)	15 (30)
EV-D68/PeV-A6	0 —	1 (3)	0 —	1 (2)
RV-A38	0 —	1 (3)	0 —	1 (2)
RV-A101	0 —	1 (3)	0 —	1 (2)
RV-A24/PeV-A6	0 —	1 (3)	0 —	1 (2)
RV-A81	0 —	1 (3)	0 —	1 (2)
RV-A54	0 —	1 (3)	0 —	1 (2)
CVA2	0 —	0 —	1 (6)	1 (2)
CVA4	0 —	0 —	1 (6)	1 (2)
CVA9	0 —	0 —	1 (6)	1 (2)
CVA16	0 —	0 —	1 (6)	1 (2)
PeV-A1	0 —	0 —	1 (6)	1 (2)
Nontyped EV/RV	0 —	2 (6)	1 (6)	3 (6)

**Abbreviations:** CSF = cerebrospinal fluid; CVA = Coxsackie A virus; PeV-A6 = parechovirus A6.

* Specimens tested at CDC laboratory.

^†^ Among EV- or RV-positive specimens.

Because some enteroviruses can cause acute flaccid limb weakness, and there was a temporal association with AFM and a nationwide severe respiratory outbreak of EV-D68 in 2014 (*2*), CDC performs EV/RV testing in an effort to identify etiologies for AFM cases. Despite a subsequent peak of AFM in 2016 (https://www.cdc.gov/acute-flaccid-myelitis/afm-surveillance.html), CDC did not receive reports of large outbreaks of severe respiratory illness in 2016. Further, there has been limited detection of pathogens in CSF in these cases; virus identified in CSF would be considered etiologic. Almost all patients with AFM have reported signs and symptoms consistent with viral illness in the weeks preceding limb weakness. Clinical, laboratory, and epidemiologic evidence to date suggest a viral association. CDC and collaborators continue to investigate risk factors for AFM and to study the causes and mechanisms of AFM.

Parents and caregivers are urged to seek immediate medical care for a child who develops sudden weakness of the arms or legs. In the evaluation of a child with acute flaccid limb weakness, clinicians are advised to inquire about recent fever with or without antecedent respiratory or gastrointestinal symptoms and to collect timely specimens for viral testing, including CSF, serum, respiratory, and stool specimens. Additional information for clinicians is available at https://www.cdc.gov/acute-flaccid-myelitis/hcp/index.html. Patients with acute flaccid limb weakness should be reported to their health departments as soon as possible regardless of laboratory or MRI findings.

## Footnotes

* https://www.cdc.gov/mmwr/epix/epix.html.
